# The Epidemiology of Soil-Transmitted Helminths in Bihar State, India

**DOI:** 10.1371/journal.pntd.0003790

**Published:** 2015-05-20

**Authors:** Katie Greenland, Ruth Dixon, Shabbir Ali Khan, Kithsiri Gunawardena, Jimmy H. Kihara, Jennifer L. Smith, Lesley Drake, Prerna Makkar, Sri Raman, Sarman Singh, Sanjay Kumar

**Affiliations:** 1 Faculty of Infectious and Tropical Diseases, London School of Hygiene and Tropical Medicine, London, United Kingdom; 2 Partnership for Child Development, Imperial College, London, United Kingdom; 3 Deworm the World, Washington, D.C., United States of America; 4 Patna University, Bihar, India; 5 Department of Parasitology, Faculty of Medicine, University of Kelaniya, Kelaniya, Sri Lanka; 6 Eastern and Southern Africa Centre of International Parasite Control, Kenya Medical Research Institute (KEMRI), Nairobi, Kenya; 7 London Centre for Neglected Tropical Disease Research, Imperial College London, School of Public Health, Faculty of Medicine, Norfolk Place, London, United Kingdom; 8 Division of Clinical Microbiology & Molecular Medicine, All India Institute of Medical Sciences, New Delhi, India; 9 Secretary Health-cum- Executive Director State Health Society, Bihar, India; Swiss Tropical and Public Health Institute, SWITZERLAND

## Abstract

**Background:**

Soil-transmitted helminths (STHs) infect over a billion individuals worldwide. In India, 241 million children are estimated to need deworming to avert the negative consequences STH infections can have on child health and development. In February-April 2011, 17 million children in Bihar State were dewormed during a government-led school-based deworming campaign. Prior to programme implementation, a study was conducted to assess STH prevalence in the school-age population to direct the programme. The study also investigated risk factors for STH infections, including caste, literacy, and defecation and hygiene practices, in order to inform the development of complementary interventions.

**Methods:**

A cross-sectional survey was conducted among children in 20 schools in Bihar. In addition to providing stool samples for identification of STH infections, children completed a short questionnaire detailing their usual defecation and hand-hygiene practices. Risk factors for STH infections were explored.

**Results:**

In January-February 2011, 1279 school children aged four to seventeen provided stool samples and 1157 children also completed the questionnaire. Overall, 68% of children (10-86% across schools) were infected with one or more soil-transmitted helminth species. The prevalence of ascariasis, hookworm and trichuriasis was 52%, 42% and 5% respectively. The majority of children (95%) practiced open defecation and reported most frequently cleansing hands with soil (61%). Increasing age, lack of maternal literacy and certain castes were independently associated with hookworm infection. Absence of a hand-washing station at the schools was also independently associated with *A*. *lumbricoides* infection.

**Conclusions:**

STH prevalence in Bihar is high, and justifies mass deworming in school-aged children. Open defecation is common-place and hands are often cleansed using soil. The findings reported here can be used to help direct messaging appropriate to mothers with low levels of literacy and emphasise the importance of water and sanitation in the control of helminths and other diseases.

## Author Summary

Deworming is recognised as a cost-effective way to increase the educational attainment and health of school-age children who experience high prevalence and intensity of soil-transmitted helminth (STH) infections and bear the majority of the disease burden. STH transmission is driven by a number of factors including the environment, water availability and lack of sanitation. Previous government surveys in India revealed small geographic areas have extremely heterogeneous prevalence, generating interest in exploring what explains this heterogeneity. Having recently implemented a large deworming progamme, the government of Bihar State was keen to utilise the deworming campaign to undertake complementary activities to reduce transmission and establish an integrated approach to STH control. This paper presents the results of the prevalence and intensity cross-sectional survey and takes an exploratory look at the contribution of key risk factors to transmission. Maternal literacy, caste, open defecation (a common practice) and absence of handwashing stations at schools were associated with increased risk of hookworm or ascariasis. Using this information it is possible to hypothesise about potential key areas of focus during the deworming campaign and interventions that could complement deworming programmes, as well as to develop some key messages to accompany the next deworming programme in Bihar.

## Introduction

Soil-transmitted helminths (STHs) are among the most common chronic infections worldwide, estimated to infect over a billion individuals in tropical and subtropical regions, mainly in low and middle income countries [1]. A number of intestinal worms fall under the STH umbrella: the roundworm *Ascaris lumbricoides*, whipworm *Trichuris trichiura* and two species of hookworm, *Ancylostoma duodenale* and *Necator americanus* [2]. STHs are predominantly transmitted when faeces containing eggs are deposited into the environment, develop to an infective stage and are transmitted via ingestion or across the skin boundary (hookworm). Thus prevalence is highest in areas where hygiene is poor, safe water and sanitation facilities are lacking and health services are insufficient [1]. STH infections rarely result in death, but they affect nutrition, resulting in anaemia, loss of appetite, intestinal damage and reduced absorption of vitamin A [2, 3], impacting on growth and cognition at critical stages of a child’s development [4]. School-age children are particularly vulnerable to these negative outcomes as they harbour the greatest worm burdens [2].

Control of STHs is therefore paramount and control initiatives centre around three themes: chemotherapy, sanitation, and health promotion. Regular school-based deworming is a proven, cost-effective strategy that can avert the health and educational consequences of STH infections [5, 6]. In 2001, World Health Assembly Resolution 54.19 set endemic countries the target of ensuring that at least 75% of school-age children at risk of morbidity were receiving regular treatment by 2010 [7]. However, the global coverage of treatment in at-risk areas at the end of 2009 was 31% [8]. Successfully implemented sanitation interventions and effective health promotion can reduce exposure to faecal material and thereby may impact substantially on helminth transmission [9, 10]. As deworming programmes are scaled up and sustainable methods of delivering treatment are established, there is renewed focus to determine how sanitation and behaviour change messages can best complement deworming activities.

In India, one of the most populous of the high burden endemic countries [11], an estimated 241 million children are in need of deworming [12]. Bihar State in eastern India is one of the most populated states with some of the lowest development indicators [13]. Prior to implementation of a school-based deworming programme between February and April 2011, the State Health Society Bihar conducted two sets of surveys to determine the geographical distribution of STH infections in this region. These surveys aimed to inform the state treatment strategy and to provide comprehensive epidemiological baseline data for future programme evaluation. Previous surveys conducted in Bihar have revealed large spatial heterogeneity in STH prevalence and it was hypothesised that caste could play a role in explaining this heterogeneity and may be a factor to consider epidemiologically when determining treatment strategies. In Hindu society the caste system divides individuals into social strata that determine privileges and limitations that are inherited from one generation to the next. Although now abolished, the caste system is an engrained part of society in some places, including Bihar. A person’s caste can define not only socio-economic status but also type of job [14], aspects of diet and possibly some behavioural characteristics too. The government released data collected during the large prevalence surveys to allow additional exploration of caste, socioeconomic and behaviour-related risk factors for helminth infections to try and understand influencing factors in heterogenous prevalence areas. The aim of the analysis here was to report the determined prevalence and intensity of STH in Bihar and to inform ongoing programmatic efforts to support deworming with behaviour change and sanitation interventions.

## Methods

### Study design and setting

Bihar state is land-locked, bordered to the north by Nepal and split by the river Ganges which flows from west to east through the state. Located far from the sea and influenced by the Himalayas, the state has a continental monsoon climate, typical of most of Northern India.

The survey conducted in Bihar State was a school-based, cross-sectional survey. Data on Bihar is scarce; according to recent reports, Bihar still has high levels of poverty and low levels of basic sanitation: 33% of the population falls into the lowest wealth quartile in India, only 17% of households have access to a toilet facility and 12% of children report having had diarrhoea in the last week [15]. Bihar is composed of thirty-eight administrative districts, four of which were selected for the survey: Araria, Aurangabad, Muzaffarpur and Gopalganj. These districts were selected to complement the existing survey data in Bihar which was collected in Supaul and Patna in 2010 (not reported here).

### Study population and sampling

Primary school children were surveyed using a two-stage cluster sampling scheme: five primary schools were randomly selected in each district, within which sixty-five children were selected using a table of random numbers and the school’s list of registered children (in attendance) as the sampling frame. The children were selected class by class, spread equally between year groups and half male and half female. Any children younger than four were excluded even if they were enrolled at the school. A different random number table was provided for each school, class and for males and females. Children in a class were asked to stand and then counted out to the specifications of the random number table. The selection procedure took non-compliance into account and aimed to ensure that a minimum of fifty children were sampled per school. This sample size and sampling methodology is deemed sufficient for determining prevalence in a district, taking into account the design effect and misclassification errors [16, 17].

### Data collection

All laboratory technicians underwent a three-day training to ensure study methodologies were consistent. They were divided into two teams, and employed to collect the data over a period of three weeks completing one district each week. Prior to the study, an information letter was sent by the state health department inviting the school to participate. Data were collected from each school over the course of two days. On day one, the team spoke with teachers and parents, ensured receipt of the letter, answered questions and took informed consent from the head teacher of each school. Children were selected for the study, provided with a small screw capped plastic container, other sample collection materials and a local language flyer explaining the study verbally and pictorially. School coordinates were recorded using a hand held Global Positioning System Monitor (GPS). Students collected their samples at home the next morning and brought them to a collection point that day. Samples were discarded if the ID labels did not match the child’s name on the registration list. Participants also provided basic demographic data including age, sex, caste and class. In collecting information on caste local terms and broad classifications were pre agreed and used as follows: General Caste; Backward Caste One; Backward Caste Two; Extremely Backward Caste; Scheduled Caste) and Scheduled Tribes [18]. Muslim children were not classified by caste as it was difficult to establish standard classifications that would be reliable and universally applicable—the caste system is mainly associated with Hinduism. Each child was interviewed in the local language by a trained community volunteer using a short questionnaire which included additional socio-demographic questions and inquired into defecation and hygiene practices. Survey teams also recorded information on the presence of water on tap, hand-washing stations and toilets on the school premises and documented any known recent deworming activities including the State community based Lymphatic Filariasis mass drug administration (MDA) which included Albendazole.

### Laboratory methods

All stool samples were processed within a few hours of collection using the modified Kato-Katz technique (kits supplied by Vestergaard-Frandsen) recommended by WHO for use in field settings [19]. Screening of infection for STH was based on a double Kato-Katz smear of 41.7 mg prepared from fresh stool samples. Samples were left to clear for a minimum of 20 minutes and examined within one hour of preparation since hookworm eggs would be cleared thereafter. The mean total number of eggs was expressed as eggs per gram (EPG) of faeces. Senior parasitologists carried out quality control on every negative slide and confirmed the first egg identification on each slide under the microscope.

### Statistical analysis

Egg counts were multiplied by 24 to adjust them to eggs per gram and classified as ‘light’, ‘medium’ or ‘heavy’ intensity infections according to WHO guidelines [16]. Prevalence statistics were used to produce maps in Arc Map 9.3 (ERSI, Redlands, CA, USA). Questionnaire data were double entered into MS Excel, and any missing or unclear data were clarified with the child during sample collection. The data were cleaned and transported to Stata 12 for analysis (StataCorp, 2011). Variables detailing the frequency (always, sometimes or never) of use of different defecation sites (open field, jungle, river or latrine) were recoded to create one variable for defecation practice. One variable for hand-hygiene practices was similarly created from questions on whether ash, soil, water only, or soap are ever used to wash hands after defecation. Due to data recording issues, analysis of defecation practices was restricted to Aurangabad and Gopalganj districts only. Castes are described in the categories outlined in Table 1 (Castes 1 to 6). As only twelve children from Caste 6 were included in the survey, they were combined with children from Caste 5 to allow for stratification by caste in further analysis. The relationship between STH infection and demographic and socio—economic variables (household assets), maternal education (proxy based on maternal literacy), caste, defecation practice, hand-hygiene practice, and school-level variables was explored in univariate analysis, taking into account the cluster effect of school. A log binomial model (family: binomial, link: log) was developed to allow for and to explore between-school variance in infection levels, assuming a fixed effect of individual and school level variables and using generalised estimating equations to account for school-level clustering. Explanatory variables significant at *P* < 0.2 level in univariate analysis were included in the model, which looked for associations significant at *P* < 0.05.

**Table 1:** Local caste classification and adopted categories used to describe castes.

Local Caste Classification	Adopted Caste Categories
**General Caste**	Caste 1
**Backward Caste One**	Caste 2
**Backward Caste Two**	Caste 3
**Extremely Backward Caste**	Caste 4
**Scheduled Caste**	Caste 5
**Scheduled Tribe**	Caste 6

### Ethics

The study was carried out by the State Government of Bihar in order to inform a public health programme. Data for the risk factor analysis was released by the Government and has been analysed and published only with their permission. Ethical consent was sought from Imperial College Ethical Review Board for the publishing of the data (June 2011); the board returned that no ethical approval was required as the data collection was not primarily research but for a public health programme, and the government had authorised publication of its public health information.

Consent procedures were in line with those laid out by the government of Bihar. Letters were sent in advance to schools to allow them sufficient time to liaise with parents and decline consent. On the survey day, as many parents as possible were invited to the schools on survey day to observe the distribution of sample pots. No samples were requested from children on the day, but they were sent home each with the required materials and a flyer explaining the survey and instructions for collecting the sample. Children who presented with a sample the next morning were assumed to have parental consent to participate in the study. No information was retained or utilized for any child who did not return a sample. Informed consent was taken from each head teacher on behalf of the parents before the materials were distributed to children, and the school head teachers signed a consent form as a written record of informed consent. Verbal assent was taken from every child. Schools were provided with the results of the survey the next day, which were delivered and explained by a member of the survey team. All schools in Bihar were provided with deworming medication as well as two trained teachers during state deworming.

## Results

### Characteristics of study participants

Between January and February 2011, a total of 1279 children from 20 schools completed the survey questionnaire, of whom 1157 (90%) produced a viable faecal sample and were included in further analysis. Study participants were registered in Classes 1 to 8 and aged 4 to 17 years (median = 9 years); 52% were female. Twenty-six percent of children were Muslim. The remaining children (74%) were Hindu and classified as Caste 1 (4%), Caste 2 (17%), Caste 3 (24%), Caste 4 (8%) and Caste 5 or 6 (22%). Age and sex distributions between schools were comparable, but caste composition varied by school (Bartlett's test for equal variances: χ^2^ = 209.54, degree of freedom = 19, *P* <0.001). Eighty-one percent of children (897/1118) belonged to households that owned a phone (mobile or landline). Overall 14% (149/1099) of households did not own any mode of transport, while three-quarters (n = 825) owned a bicycle and 11% owned a motorbike. According to the 2011 Census, 41% of Bihari households own a bicycle and 4% have a motorbike [20], suggesting that children who are in school may come from more affluent families than the average Bihari. Only 26% (295/1150) of children had literate mothers, lower than the estimated female literacy rate in Bihar state (2001 = 33%, 2011 = 53%)[21]. Literacy and ownership of household assets were closely correlated with caste. Thirty-one percent of children (361/1149) attended school barefoot on the survey day.

### Soil-transmitted helminth infection

**Fig 1:**
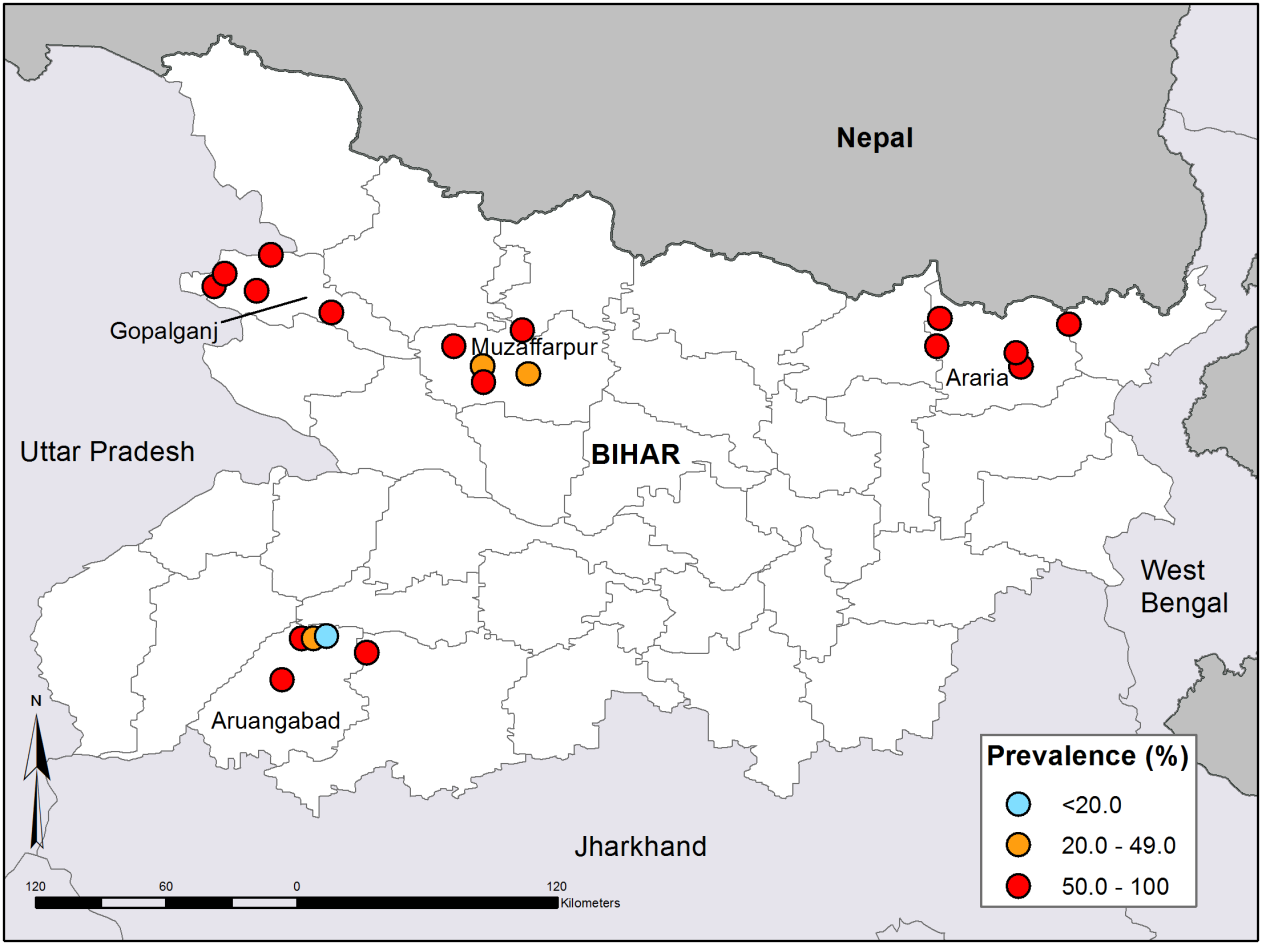
Prevalence of soil-transmitted helminths in the 20 schools in Bihar.

The overall prevalence of any STH infection was 68%, ranging from 10% to 96% across schools (Fig 1). Prevalence varied by district: Aurangabad 49%; Muzaffarpur 62%; Araria 80% and Gopalganj 82% (Table 2). All surveyed districts exceeded the WHO-defined prevalence threshold for mass distribution of anthelmintic drugs. *A*. *lumbricoides* was the most prevalent infection (52%), followed by hookworm species (42%) and *T*. *trichiura* (5%). Between a third and a half of infected children in each district harboured more than one helminth species, but triple infection was rare (Table 2). *T*. *trichiura* and hookworm infections were almost all light intensity (52/54 and 475/483 infections for *T*. *trichiura* and hookworm respectively). Five hookworm infections and 17% (n = 102/600) of *A*. *lumbricoides* infections were of moderate intensity. Heavy intensity infections were only found in three children, all of whom were infected with hookworm. Medium and heavy intensity hookworm infection (n = 8) were found in Aurangabad and Gopalganj in the West of the state, while the majority (69.6%) of the 102 medium intensity *A*. *Lumbricoides* infections were found in one district: Araria, in the North East.

**Table 2:** Prevalence of soil-transmitted helminth infections among primary school children in the four surveyed districts (N = 1157).

	Number and (%) of children found positive per district*				
	Overall (N = 1157)	Araria (N = 300)	Aurangabad (N = 322)	Muzaffarpur (N = 330)	Gopalganj (N = 327)
***A*. *lumbricoides***	600 (51.9)	191 (72.1)	74 (25.0)	137 (45.7)	198 (66.9)
***T*. *trichiura***	54 (4.7)	30 (11.3)	5 (1.7)	15 (5.0)	4 (1.4)
**Hookworm**	483 (41.8)	86 (32.5)	119 (40.2)	116 (38.7)	162 (54.7)
**Any Infection** ^	786 (67.9)	211 (79.6)	145 (49.0)	187 (62.3)	243 (82.1)
**Total Single Infection**	456 (58.0)	128 (60.7)	94 (64.8)	111 (59.4)	123 (50.6)
***A*. *lumbricoides* & *T*. *trichiura*** ^#^	16 (5.2)	8 (11.4)	0	7 (9.9)	1 (0.8)
***A*. *lumbricoides* & hookworm** ^#^	287 (92.9)	61 (87.1)	47 (95.9)	62 (87.3)	117 (98.3)
***T*. *trichiura* & hookworm** ^#^	6 (1.9)	1 (1.4)	2 (4.1)	2 (2.8)	1 (0.8)
**Total Double infection**	309 (26.7)	70 (33.2)	49 (33.8)	71 (38.0)	119 (49.0)
**Triple Infection**	21 (1.8)	13 (6.2)	2 (1.4)	5 (2.7)	1 (0.4)

* denominators vary due to missing data

^^^ refers to number of children positive for at least one species of soil transmitted helminth

^#^ Percentages calculated using the total number of children with double infections

### Personal hygiene

Overall, 208 children (18% of all participants) had access to a latrine at home and a further 47 (4%) reported having access to a community toilet facility. The majority of children practiced open defecation all (63%), or some (33%) of the time, of whom almost all usually reported defecating in fields as opposed to a river or the jungle (n = 484, 2 and 8 respectively). Boys more frequently reported open defecating than girls (91% vs. 84%, P <0.001). Defecation practices also varied by caste: 44% of children from Caste 1 sometimes or always defecated in the open, compared with almost all (88.8%) children from other castes (*P* <0.001). Post-defecation hand-hygiene usually involved the use of soil and soap (61% and 36% respectively), while a small minority of participants reported using ash (1.9%) or water only (0.5%). Soap use also varied by caste: 53% (25/47) of children from Caste 1 reported exclusive use of soap for hand cleansing compared with 21% (167/799) of children from Castes 2 to 6, and 17% (49/292) of Muslims (*P* <0.001). Soil usage varied similarly by caste. Hygiene practices were closely-related to defecation practices: latrine users were almost three times more likely to state that they use only soap to cleanse their hands than children who sometimes or always defecate openly (52% vs. 17%, *P* <0.001).

Teachers from three schools recalled receiving prior anthelminthic treatment inclusive of that received as part of the Lymphatic Filariasis programme. Of the 20 schools, 15 had latrines, 11 had hand-washing stations (usually a hand pump that was unlocked and had water in it) and two had water on tap. Nine of the 11 hand-washing stations were found in the schools in Gopalganj and Aurangabad districts.

### Risk factors for infection

Potential risk factors for STH infection were explored. *T*. *trichuris* was dropped due to low numbers of infections. The main findings are summarised in Table 3. There was no sex difference in the risk of either infection. Older children (over 12 years) were more likely to be infected with hookworm than children aged 4 to 7 (RR = 1.3, 95%CI 1.0–1.6), but there was no evidence that the prevalence of *A*. *lumbricoides* infection varied with age. Ownership of a phone or motorbike, both indicative of greater household wealth, were protective against hookworm infection when compared with children without a phone or any means of transport (phone ownership: RR = 0.9, 95%CI 0.8–0.9; motorbike: RR = 0.6, 95%CI 0.4–0.9). Children from households with motorbikes were also less frequently infected with *A*. *lumbricoides* (RR = 0.9, 95%CI 0.7–1.0). Children with literate mothers (proxy for maternal education) were at reduced risk of both infections, particularly hookworm (RR = 0.5, 95%CI 0.4–0.7) (Table 3). Household assets and maternal literacy were closely associated and only maternal literacy was retained during later analysis. Results also suggest that belonging to a caste other than Caste 1 or being Muslim might be associated with risk of hookworm infection (Table 3). Attending school barefoot did not increase the risk of either hookworm or *A*. *lumbricoides* infection (hookworm: 41% and 43%, *P* = 0.561; *A*. *lumbricoides*: 52% and 52%, *P* = 0.925 for children with and without shoes respectively). Children who solely use latrines were considerably less likely to be infected with hookworm than children who always open defecate (13% vs. 49% respectively, *P* <0.001); however, latrine usage was not found to be associated with *A*. *lumbricoides* infection (Table 3). Soap use appeared protective against hookworm and *A*. *lumbricoides* infection when compared with other methods of hand-cleansing after defecation (mainly use of soil), although these associations had only borderline significance (Table 3). Soap use at this juncture is indicative of soap use in general even though children do not acquire infection directly from hands contaminated at this time.

**Table 3:** Risk factors for hookworm and *A*. *lumbricoides* infection, Bihar India (N = 1157).

			Hookworm					*Ascaris lumbricoides*				
				Univariate		Multivariate			Univariate		Multivariate	
		N	% infected	OR (95%CI)	*P*	Adj. OR (95%CI)	*P*	% infected	OR (95%CI)	*P*	Adj. OR (95%CI)	*P*
**Gender**												
	Female	600	40.7	ref.		ref.		52.2	ref.		ref.	
	Male	557	42.9	1.1 (0.9–1.4)	0.186	1.0 (0.9–1.3)	0.456	51.5	1.0 (0.9–1.0)	0.301	1.0 (0.9–1.0)	0.264
**Age group**												
	4–7 yrs	308	40.3	ref.		ref.		56.2	ref.			
	8–11 yrs	590	42.5	1.2 (0.9–1.5)	0.157	1.2 (1.0–1.5)	0.085	50.2	1.0 (0.9–1.2)	0.511	1.0 (0.9–1.2)	0.496
	12–17 yrs	259	41.7	1.3 (1.0–1.6)	0.029	1.3 (1.0–1.7)	0.030	50.6	0.9 (0.8–1.1)	0.438	1.0 (0.8–1.1)	0.578
**Caste**												
	Caste 1	47	34.0	ref.		ref.		34.1	ref.		ref.	
	Caste 2	200	46.0	1.5 (0.9–2.6)	0.143	1.2 (0.8–1.7)	0.451	56.0	1.4 (0.8–2.5)	0.214	1.3 (0.8–2.3)	0.281
	Caste 3	272	37.9	1.8 (1.1–2.8)	0.017	1.3 (1.0–1.6)	0.036	36	1.5 (0.8–2.7)	0.184	1.4 (0.8–2.5)	0.213
	Caste 4	89	52.8	1.5 (0.9–2.4)	0.104	1.1 (0.8–1.4)	0.694	64	1.7 (0.9–3.2)	0.103	1.6 (0.9–2.8)	0.143
	Castes 5 + 6	249	44.2	1.8 (1.2–2.7)	0.005	1.4 (1.0–1.8)	0.031	54.2	1.5 (0.7–3.1)	0.273	1.4 (0.7–2.8)	0.345
	Muslim	294	38.1	1.5 (1.1–2.0)	0.004	1.2 (1.0–1.4)	0.121	61.6	1.6 (0.9–3.0)	0.112	1.5 (0.9–2.7)	0.136
**Maternal education**												
	Illiterate	855	44.1	ref.		ref.		55.8	ref.		ref.	
	Literate	295	34.6	0.5 (0.4–0.7)	<0.001	0.8 (0.7–1.0)	0.032	40.7	0.9 (0.8–1.0)	0.081	0.9 (0.8–1.1)	0.263
**Defecation practice**												
	Open defecation only	368	48.9	ref.		ref.		46.7	ref.			
	Open defecation and latrine	192	47.9	0.9 (0.7–1.2)	0.567	1.0 (0.8–1.3)	0.895	46.4	0.9 (0.8–1.1)	0.236		
	Latrine only	23	13	0.4 (0.2–1.0)	0.042	0.5 (0.2–1.1)	0.097	30.4	0.8 (0.7–1.1)	0.164		
**Hand-hygiene practice**												
	Soil, ash or water only	591	40.1	ref.		ref.		55.7	ref.		ref.	
	Soap and ash/soil/water	311	49.5	0.9 (0.8–1.0)	0.010	1.0 (0.9–1.1)	0.355	53.4	1.1 (1.0–1.2)	0.184	1.1 (1.0–1.2)	0.025
	Soap only	242	34.3	0.8 (0.6–1.0)	0.099	0.9 (0.7–1.3)	0.625	41.3	0.9 (0.8–1.0)	0.084	0.9 (0.8–1.1)	0.236
**Hand-washing station at school**												
	No	517	40.2	ref.				65.2	ref.		ref.	
	Yes	640	42.9	0.8 (0.6–1.2)	0.271			41.1	0.6 (0.4–0.9)	0.025	0.6 (0.4–0.9)	0.016

RR = risk ratio, 95%CI = 95% confidence interval, P = p value. Univariate analysis takes account of clustering within schools. The model was restricted to two districts (Aurangabad and Gopalganj) due to data collection errors for some variables in the other districts.

For caste classification see Table 1. Castes 5 and 6 were combined for analysis due to small numbers. Muslims were excluded from the caste classification and are therefore presented collectively and separately.

Maternal education (literacy) was assessed by asking the child whether their mother can read their hindi textbook. The proportions of literate mothers were similar for muslims and hindus, even in urdu speaking schools. All responses are therefore included in analysis.

Defecation practice: variable created from questions pertaining to frequency (usually, sometimes, never) of use of an open field, river, jungle or latrine for defecation. Almost all open defecation (98%) takes place in an open field.

Hand-hygiene practice: 95% of individuals within the "soil, ash or water" category use only soil to hand-wash after using the toilet.

After adjustment in the multivariable model (see Table 3 for variables included in the model), increasing age, lack of maternal literacy and Castes 3, 5 and 6 remained significantly associated with hookworm infection. Absence of a hand-washing station at the schools was independently associated with *A*. *lumbricoides* infection after adjusting for gender, age, caste, maternal literacy and hand-hygiene practice.

## Discussion

The survey revealed high prevalence of STHs in all four districts under study. Hookworm and *A*. *lumbricoides* infections were widespread, but *T*. *trichiura* was rarely detected, probably due to the high temperatures in Bihar during certain times of the year that may not allow the full development of *T*. *trichiura* eggs. Together with survey data from two additional states, point-level environmental data (annual rainfall and temperature) and socio-economic data (literacy at district level), the state-wide prevalence of STHs was modelled. The modelled prevalence data resulted in the government decision to treat the whole state with anthelmintics twice a year, integrating school based deworming with the Lymphatic Filariasis programme which includes Albendazole. Between February and April 2011, a school-based deworming in Bihar’s 67,000 government schools reached 17 million school-age children, including one million un-enrolled children, of the targeted 21 million, one of the largest school-based deworming programmes globally to date (www.dewormtheworld.org). A second round of treatment is planned in 2012. The data collected in this survey will provide a useful baseline of prevalence and intensity of STH infection, as well as detailed contextual information. Both of which are valuable for future measurement of the impact of the deworming programme and will form part of the state’s monitoring and evaluation programme.

This paper further describes behavioural, socio-economic (based on a proxy of literacy in the model) and caste-related factors found to be associated with STH infection, useful for informing campaigns accompanying deworming activities in the Bihar context. Our findings on each of these topics are considered in turn. Behaviour is a key factor in determining exposure to infective stages and consequently affects the transmission and reinfection rate. As with other studies [22–24], we found that open defecation increased the risk of hookworm infection. Defecation fields are likely to be highly-contaminated environments with high rates of transmission, particularly as hookworm larvae can directly penetrate the skin and children often go around barefoot. We did not identify the absence of footwear as a risk factor for infection as some other studies have shown [25], possibly because all children spend some time barefoot and are similarly exposed, because the majority of shoes are open shoes such as flip flops and might afford little protection, or because transmission is not only via this route: *N*. *americanus* can penetrate other skin surfaces and *A*. *duodenale* can be transmitted orally. Shoe campaigns may not be the way forward. Unlike hookworm, *A*. *lumbricoides* is only transmitted via ingestion of infective stage eggs; and in concordance with this we found that open defecation was less important for transmission (being bare foot in an open defecation field would not contribute to *A*. *Lumbricoides transmission*). We could expect that behaviours which increase the likelihood of ingesting eggs play more of a role. We have some evidence at univariate level that soap use protects against infection and that soil use is a risk factor. The latter may be because the soil is infected with infective eggs if taken from an area close to the defecation site. Self-reported measures of hand-washing tend to overestimate soap use [26], so it is possible that hand-washing with soap actually takes place too infrequently to reduce risk in highly-contaminated environments. We are not able to firmly conclude on the role of hand-washing with soap or the risk posed by using soil for hand-cleansing. Given the “soil-mouth” transmission route, it is plausible that cleaning hands with soil in defecation fields contaminated with infective stage eggs (if soil is taken from the surrounding area) could be a risk factor for *A*. *lumbricoides*. A recent review found inconclusive evidence that hand-washing can reduce *A*. *lumbricoides* infection [27].

We now turn our attention to the findings pertaining to maternal literacy (our proxy for socio-economic status) and caste. It is unsurprising that defecation and hand-hygiene practices were closely related to each other; a latrine offers a more enabling environment for hand-washing with soap than an open field where soil was more commonly used for hand-cleansing. However, latrine use, and therefore relative frequency of soil or soap use, may be associated with maternal literacy. Maternal literacy was negatively associated with STH infection. This observation is concordant with other contexts [24], where persistent health differences are seen across different socio-economic groups; education is an important social determinant of health [28]. The complex, socially-stratified caste system in India, within which some castes are typically poorer and less educated than others—by our definition Caste 1 would be considered more educated and wealthy than Caste 6—plays an important role in determining health status [18]. As you are born into a caste and remain in it, some castes are trapped at a certain social level which makes it difficult to break the cycle of poverty and ill health. Defecation practice, hand-hygiene behaviour and maternal literacy—risk factors for helminth infection—all vary by caste, yet some caste differences in risk of infection remain in the adjusted model. This suggests that other intrinsic behavioural or cultural differences exist between the castes that were not captured by the indicators collected in the questionnaire. It is possible that children from different castes have different environmental exposure to helminth eggs if they live in geographically separate parts of the village and have differential exposure to faecal material. It was not possible to analyse this spatially. All castes have high prevalence of infection and there is no indication that after accounting for differences in socio-economic status (literacy) and hygiene behaviour that caste itself plays a significant role in determining prevalence in a particular area.

Water, sanitation and hygiene (WASH) behaviours are important in STH transmission and therefore WASH forms an important element of STH control alongside regular deworming. Should resources be available to support large-scale WASH and behaviour change campaigns, successfully implemented interventions could also complement school-based deworming. Such interventions could include a behaviour change campaign to accelerate the uptake and use of latrines, preceded by formative research to understand sanitation adoption behaviour and inform the intervention design [29, 30], or behaviour change campaigns to improve hand-washing practices and reduce the prevalence of cleansing hands with soil.

Increasing the coverage of (working) hand-washing stations in schools enables hand-washing to take place and increases general water use. It is interesting to see that children in schools with hand-washing stations had fewer STH infections. Although hand-washing stations can facilitate hand-washing, in this case it appears that their presence also allows use of water for other purposes which could reduce the risk of transmission. Indeed, survey teams observed that cooking utensils and plates were washed at hand pumps where available, but using soil—which could be contaminated with eggs—in their absence. Available water also makes the practice of washing fruit, vegetables and other food picked from the ground feasible. However, the relative importance of different transmission routes (e.g. unwashed food) is currently unknown and should be further quantified.

Our study has some limitations. Although Kato Katz is the recommended methodology for this type of survey, the technique is known to have less than perfect sensitivity as a diagnostic test especially at lower intensity infections and is limited by taking only one days sample rather than consecutive days when egg excretion is known to fluctuate. The actual prevalence of infection could therefore be higher than identified. One of the surveyed districts, Aurangabad, was a second choice district in this area because the first district selected could not be surveyed for security reasons. The risk factor analysis is restricted to the measured variables and may not present a complete picture of risk patterns and dynamics at play. Only three schools remembered the LF deworming programme despite extensive probing. Although we cannot discount the possibility that the reach of the LF programme was larger than this study indicated, we conclude that few children would have received Albendazole at this time, and even if they had, there was sufficient time for reinfection to take place before the survey and prevalence is unlikely to be underestimated for this reason. As previously mentioned, defecation and hygiene practices described are based on self-report; therefore it is possible that the frequency of “good” practice has been overestimated. Given that school-children answered the questionnaire, it was not possible to better define socio-economic status, but more information on household assets, parental occupation and income could have been useful. Only 4% of the survey population were from the Caste 1. Although current data is not available on the caste composition of Bihar state, the 1931 Census found that the “Upper Castes” represented 13.7% of the population, [31] and it is possible that Caste 1 has been under-represented in this survey.

STH prevalence in Bihar is high and justifies mass treatment. This survey provides a baseline for the monitoring and evaluation process for the Bihar State School Based Deworming Programme; a paper on lessons learnt from deworming over 17 million children is under consideration (Drake et al.). In Bihar, open defecation is common-place and hands are often cleansed using soil which may be contaminated with STH eggs in open defecation fields. Maternal literacy appears to have the biggest influence on risk of infection. Caste differences exist in the prevalence of different risk behaviours and caste appears to be associated with helminth infections. However, prevalence of infection is high in all castes and caste would not be a relevant targeting tool for treatment strategies or complementary campaign activities. The findings reported have been used to help direct the health and behaviour messaging being developed as part of ongoing school based deworming campaigns in Bihar. Additionally, the results hypothesise control of helminths as an important potential benefit of WASH programmes in this context. Community awareness campaigns and health and behaviour messaging around deworming programmes should consider the low levels of female literacy and develop pictorial or visual messages as appropriate.

## Supporting Information

S1 Text Bihar ethics approval letter. (PDF)

S1 Checklist STROBE checklist. (DOC)
