# Supplementary material for: The Epidemiology of Soil-Transmitted Helminths in Bihar State, India
Source: PLoS Negl Trop Dis. 2015 May 20;9(5):e0003790. doi: 10.1371/journal.pntd.0003790 (PMC4439147; doi:10.1371/journal.pntd.0003790)
Supplement: S1 Text — (PDF) [file pntd.0003790.s001.pdf]

Imperial College London

Faculty of Medicine
Regulatory Compliance section
Joint Research Office
Imperial College London and Imperial College Healthcare NHS Trust.
Room 510A
5th Floor Lab Block
Charing Cross Hospital
Fulham Palace Road
W6 8RF

Tel: +44 (0)20 311 0206
Fax: +44 (0)020 311 0203

Lucy.parker@imperial.ac.uk
www.icl.ac.uk/clinicalresearchgovernanceoffice

**Lucy Parker**
Research Governance Manager

23rd January 2012

Dear Sir or Madam,

**RE: The importance of socioeconomic risk factors and sanitation in the epidemiology of soil-transmitted helminths in Bihar State, India (working title)**

**Public Library of Science (PLoS)**

We are writing to confirm that following an enquiry to the AHSC Joint Research Office (JRO) for Imperial College London and Imperial College Healthcare NHS Trust, we advised the Partnership for Child Development (PCD), part of Department of Infectious Disease Epidemiology of Imperial College London, that the above mentioned paper is not considered research, and therefore does not require an ethical opinion.

The JRO reviewed the details of the above mentioned paper (as laid out in the annex of this letter), which included the analysis and publication of the Indian State Government of Bihar's data on worm prevalence in school-children, with the Head of Regulatory Compliance for the College, Gary Roper. We provide this letter as evidence of ethical consideration by PCD and confirmation of our advice.

Please direct any research compliance enquiries for this paper to Ms Lucy Parker, Research Governance Manager, JRO at the address above.

Yours sincerely

Lucy Parker
Research Governance Manager
Faculty of Medicine
Imperial College London

Imperial College London

Faculty of Medicine
Regulatory Compliance section
Joint Research Office
Imperial College London and Imperial College Healthcare NHS Trust.
Room 510A
5th Floor Lab Block
Charing Cross Hospital
Fulham Palace Road
W6 8RF

Tel: +44 (0)20 311 0206
Fax: +44 (0)020 311 0203

Lucy.parker@imperial.ac.uk
www.icl.ac.uk/clinicalresearchgovernanceoffice

**Lucy Parker**
Research Governance Manager

23rd January 2012

Dear Sir or Madam,

**RE: Evaluation of diagnostic techniques for soil-transmitted helminth surveys in Bihar State, India (working title)**

**WHO Bulletin**

We are writing to confirm that following an enquiry to the AHSC Joint Research Office (JRO) for Imperial College London and Imperial College Healthcare NHS Trust, we advised the Partnership for Child Development (PCD), part of Department of Infectious Disease Epidemiology of Imperial College London, that the above mentioned paper is not considered research, and therefore does not require an ethical opinion.

The JRO reviewed the details of the above mentioned paper (as laid out in the annex of this letter), which included the analysis and publication of the Indian State Government of Bihar's data on worm prevalence in school-children, with the Head of Regulatory Compliance for the College, Gary Roper. We provide this letter as evidence of ethical consideration by PCD and confirmation of our advice.

Please direct any research compliance enquiries for this paper to Ms Lucy Parker, Research Governance Manager, JRO at the address above.

Yours sincerely

Lucy Parker
Research Governance Manager
Faculty of Medicine
Imperial College London
